# Role of Ag_2_S coupling on enhancing the visible-light-induced catalytic property of TiO_2_ nanorod arrays

**DOI:** 10.1038/srep19754

**Published:** 2016-01-21

**Authors:** Zhengcao Li, Shan Xiong, Guojing Wang, Zheng Xie, Zhengjun Zhang

**Affiliations:** 1 State Key Laboratory for New Ceramics and Fine Processing, School of Materials Science and Engineering, Tsinghua University, Beijing 100084, China; 2 Key Laboratory of Advanced Materials (MOE), School of Materials Science and Engineering, Tsinghua University, Beijing 100084, China; 3 High-Tech Institute of Xi’an, Shanxi 710025, China

## Abstract

In order to obtain a better photocatalytic performance under visible light, Ag_2_S-coupled TiO_2_ nanorod arrays (NRAs) were prepared through the electron beam deposition with glancing angle deposition (GLAD) technique, annealing in air, followed by the successive ionic layer absorption and reaction (SILAR) method. The properties of the photoelectrochemical and photocatalytic degradation of methyl orange (MO) were thus conducted. The presence of Ag_2_S on TiO_2_ NRAs was observed to have a significant improvement on the response to visible light. It’s resulted from that Ag_2_S coupling can improve the short circuit photocurrent density and enhance the photocatalytic activity remarkably.

Recently, as the demands of industrial wastewater treatment and solar energy conversion increasing, photocatalytic technology has become one of the most popular subjects. Since the photocatalytic splitting of water by titanium dioxide (TiO_2_) electrodes was discovered by Fujishima *et al*. in 1972^1^, TiO_2_-based photocatalysis^2^,^3^,^4^,^5^,^6^,^7^,^8^ has been extensively investigated due to its outstanding properties such as strong photocatalytic activity, chemical inertness, nontoxicity and low cost. However, there are still a couple of remained problems in utilizing TiO_2_ as photocatalytic material. One is the light absorption limitation in visible light region due to the wide band-gap of TiO_2_ (3.2 eV as anatase and 3.0 eV as rutile), the other is the easy recombination of photo-electrons and holes during the photocatalytic process. Hence, some recent studies have been carried out focusing on TiO_2_ response in visible light region by different methods, which include sensitization with dyes^9^,^10^, doping TiO_2_ with metal or non-metal ions^11^,^12^,^13^, and coupling TiO_2_ with narrow band-gap semiconductors^14^,^15^,^16^,^17^,^18^.

Among these methods, coupling TiO_2_ with narrow band-gap semiconductors to form heterojunction structures shows promising effects in enhancing separation of photogenerated charge carriers and improving catalytic activity of TiO_2_^19^. Compared with bare TiO_2_, CdS-coupled TiO_2_ presents a significant improvement on photocatalytic degradation of organic pollutants in industrial wastewater under visible light irradiation^20^. Nevertheless, the potential of releasing threatening Cd element is one major limitation for the application of this photocatalytic system^21^. Therefore, some other materials^22^,^23^,^24^,^25^,^26^ (such as PbS, Ag_2_S, WO_3_, ZnO, SnO_2_, α-Fe_2_O_3_ and so forth) have been investigated, and the non-toxic Ag_2_S has a narrow energy gap of *E*_g_ ~ 1.0 eV, showing promising coupling performance with TiO_2_. This is because that Ag_2_S can facilitate charge carrier separation at the heterojunction interface^27^, and increase the system’s photocatalytic efficiency. In addition, low-dimensional nanostructures of TiO_2_ coupled with nanoparticle sensitizers are expected to exhibit desirable photocatalytic activity by facilitating the charge carriers’ transfer and reducing recombination centers^28^. In previous reports, there have been some positive results of Ag_2_S-coupled TiO_2_ nanotubes^29^ and nanorods^30^,^31^ in photovoltaic devices and water splitting areas. However, as far as we know, there are few work on the relation between the degradation efficiency and the amount of Ag_2_S on the surface of TiO_2_.

Herein, we successfully synthesized the visible-light-induced TiO_2_ nanorods arrays (NRAs) coupled with different amounts of Ag_2_S nanoparticles (NPs) by using the successive ionic layer absorbance and reaction (SILAR) method. The TiO_2_ NRAs were obtained by oxidation of Ti NRAs, which were fabricated by electron beam deposition. The optimal as-prepared ultra-thin films with thickness of ~160 nm have shown pronounced short circuit photocurrent density improvement and remarkable photocatalytic activity enhancement compared with bare TiO_2_ NRAs under visible light.

## Results

### Characterization of Ag_2_S-coupled TiO_2_ NRAs

**Figure 1:**
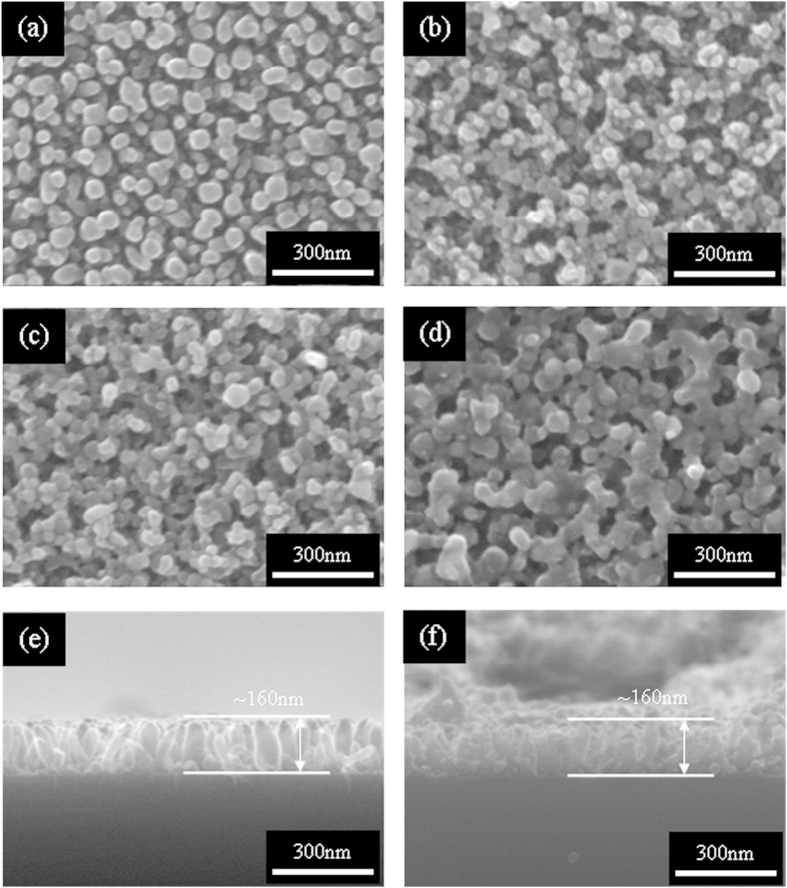
Top view SEM images of (**a**) bare TiO_2_, and different Ag_2_S-coupled TiO_2_ NRAs by SILAR method for (**b**) 20 cycles, (**c**) 30cycles, (**d**) 40 cycles; and side view of (**e**) bare TiO_2_ and (**f**) Ag_2_S-coupled TiO_2_ NRAs for 20 cycles.

Figure 1a shows the typical top view SEM images of the bare TiO_2_ film (before SILAR, 0 cycle). It can be clearly seen that TiO_2_ film consists of separate nanorods with an average diameter of 50 nm. After depositing Ag_2_S for 20, 30 and 40 cycles, the typical top view SEM images are shown in Fig. 1b–d, respectively. Without destructing the ordered TiO_2_ NRAs structure, Ag_2_S NPs were successfully deposited on the surface of TiO_2_ NRAs with different diameters from 20 to 40 cycles and the deposition amount can be readily controlled by SILAR cycles. The side view of bare TiO_2_ shows uniform NRAs with an average length of ~160 nm, as shown in Fig. 1e. As a representative example, the side view SEM image of 20-cycle sample is also given in Fig. 1f, which shows the length of the fabricated Ag_2_S-coupled TiO_2_ NRAs are ~160 nm as well.

**Figure 2:**
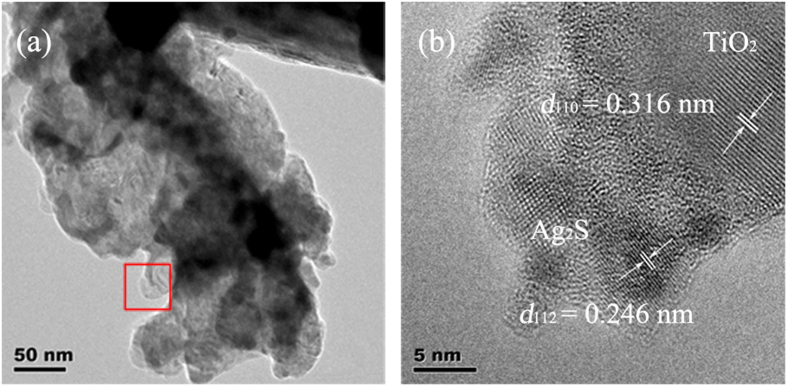
(**a**) TEM image and (**b**) the corresponding HRTEM image of Ag_2_S-coupled TiO_2_ NRAs by SILAR method for 35 cycles, corresponding to the rectangular region in (**a**).

Further observation of Ag_2_S-coupled TiO_2_ NRAs (35-cycle sample) has been confirmed by the high resolution transmission electron microscope (HRTEM) (shown in Fig. 2). The observed lattice distance of 0.316 nm and 0.246 nm corresponded to the (110) atomic planes of rutile TiO_2_ and (112) atomic planes of Ag_2_S, respectively. The HRTEM image also confirms Ag_2_S nanocrystallines (with a diameter of 5 to 20 nm) coating on TiO_2_ NRAs, as shown in Fig. 2.

**Figure 3:**
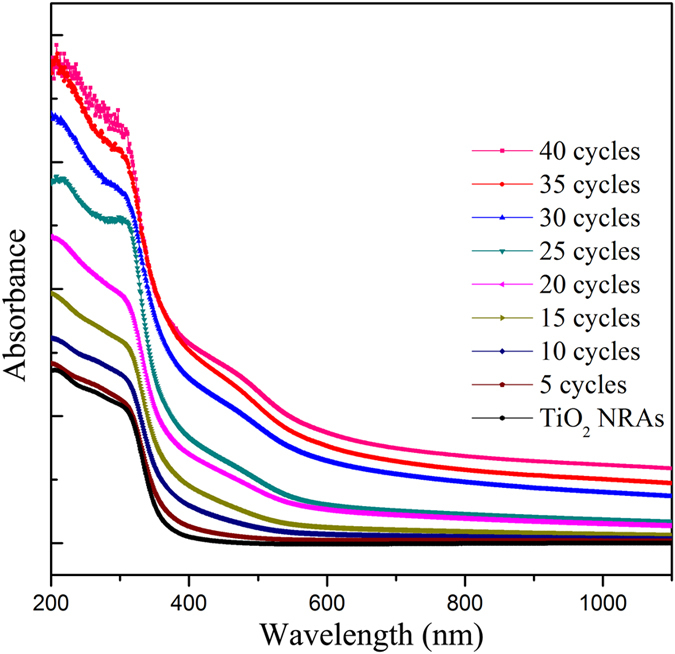
UV-visible absorption spectra of TiO_2_ NRAs and Ag_2_S-coupled TiO_2_ for various cycles.

Figure 3 displays the UV-visible absorption spectra of the Ag_2_S-coupled TiO_2_ NRAs with different SILAR cycles (*n*), from 0 to 40 cycles, respectively. For bare TiO_2_ NRAs (0 cycle), there is a clear absorption enhancement in the wavelength of 390 nm, corresponding to the bandgap of anatase (3.2 eV). Comparing the spectra of the samples with different cycles, it can be seen that the absorption of Ag_2_S-coupled TiO_2_ NRAs displays an evident enhancement in the visible-light (~400–600 nm) region from 5 to 40 cycles. Meanwhile, the absorption of the samples shows red shifts with increasing cycles. This means the band gap of the samples has been reduced as increasing the amount of Ag_2_S deposited on TiO_2_ NRAs. With Ag_2_S deposited on TiO_2_ NRAs, the absorption spectra are successfully extended to visible light region. In addition, the evident gradual enhancement of absorption implies that the amount of Ag_2_S increases with increasing depositing cycles. Therefore, compared with bare TiO_2_, a better photochemical performance under visible light irradiation is expected in Ag_2_S-coupled TiO_2_ NRAs.

### Visible-light-induced photoelectrochemical performance

**Figure 4:**
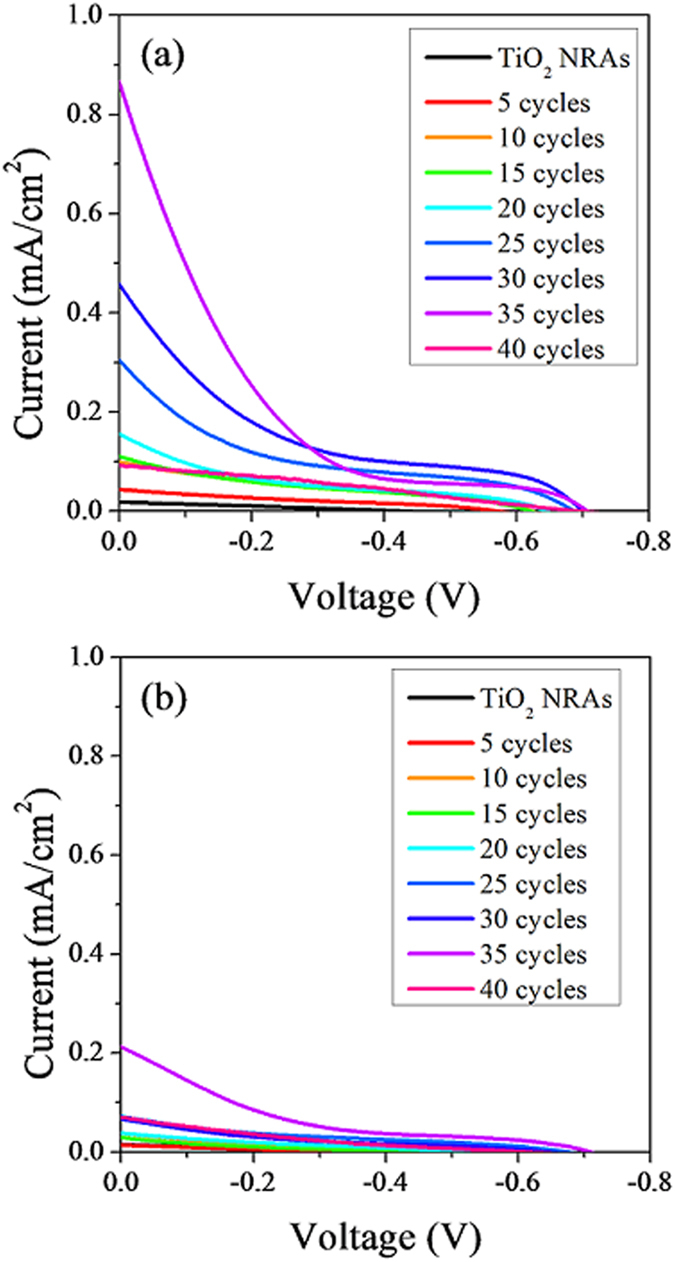
Photocurrent density-voltage curves of TiO_2_ NRAs and Ag_2_S-coupled TiO_2_ for various cycles under (**a**) visible light irradiation and (**b**) dark condition.

To further investigate the electrochemical activity under visible light, *J-V* characteristics curves (Fig. 4) of the samples from 0 to 40 SILAR cycles were measured under dark condition and visible light (illuminated by a 300 W Xe lamp at 130 mW/cm^2^, with UV light excluded by an ultraviolet cutoff filter), respectively. No significant photocurrent density was observed in the dark for all samples within the applied voltage range. While there were obvious photocurrent increases under visible-light illumination for all Ag_2_S-coupled TiO_2_ NRAs samples. It is also clear that compared with bare TiO_2_ NRAs, Ag_2_S-coupled TiO_2_ NRAs samples show much higher photocurrent densities, indicating an enhanced separation and longer lifetime of photogenerated charge carriers. Moreover, it is noteworthy that with increasing cycles, the photocurrent densities increased from 5 cycles to 35 cycles, then decreased from 35 to 40 cycles, suggesting that there is an optimum amount of Ag_2_S deposited on TiO_2_ NRAs in terms of photoelectrochemical performance.

Based on the *J-V* characteristics curves, the photovoltaic properties parameters of these samples are listed in Table 1. From 0 (bare TiO_2_ NRAs) to 35-cycle, all the electrochemical parameters increase at first, and then decrease from 35 to 40 cycles. This result is in accordance with the previous observation and confirms that with proper amount of Ag_2_S NPs decorating TiO_2_ NRAs the visible-light-induced photoelectrochemical activity is enhanced due to more effective separation of photogenerated electrons and holes. Overall, the electrochemical parameters reached a maximum value at 35 SILAR cycles. Further increase of Ag_2_S leads to deterioration of performance, since an excess of Ag_2_S NPs may destroy the one-dimensional structure of TiO_2_ NRAs due to the aggregation around the nanorods, therefore impeding effective charge transportation. Similarly, a large amount of Ag_2_S NPs may form more nanoclusters, which can become electron-hole recombination centers, causing unfavorable charge recombination and decreasing photoelectrochemical activity.

To evaluate the relative performance of samples with the optimized amount of Ag_2_S, we can compare the results with bare TiO_2_ NRAs. The 35-cycle-Ag_2_S-coupled TiO_2_ NRAs sample possesses the highest value of short circuit current density (*J*_sc_), open circuit voltage (*V*_oc_) and photovoltaic conversion efficiency (*η*), which is 6.256 mA/cm^2^, 0.707 V and 0.054%, respectively. These electrochemical parameters reach 101, 1.68 and 26.5 times improvement more than bare TiO_2_ NRAs, respectively. This suggests a pronounced enhancement of visible-light photoelectrochemical performance. Considering the length scale of nanomaterial, the 35-cycle sample shows promising absolute values of these properties.

### Visible-light-induced photocatalytic performance

**Figure 5:**
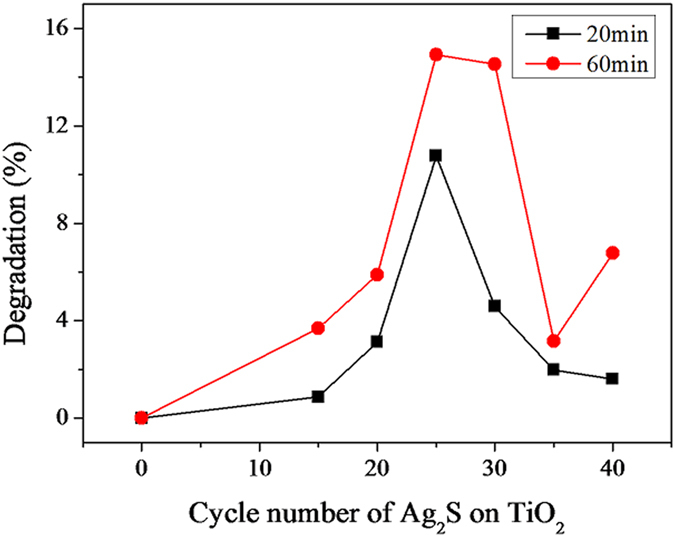
Photocatalytic degradation rates of MO under visible light irradiation using TiO_2_ NRAs (0 cycle) and Ag_2_S-coupled TiO_2_ for various cycles for 20 min and 60 min, respectively.

The photocatalytic activities of Ag_2_S-coupled TiO_2_ NRAs were explored by the degradation rate of methyl orange (MO) under visible light irradiation. Figure 5 shows the degradation percentage of MO solution degraded by bare TiO_2_ (0 cycle) and Ag_2_S/TiO_2_ NRAs with various cycles under visible-light irradiation times of 20 and 60 min, respectively. In general, TiO_2_ NRAs display a lower photodegradation rate of MO under visible-light irradiation. It is noteworthy that the degradation rate of MO obviously increases with the existence of certain amount of Ag_2_S NPs on TiO_2_ NRAs, and the 25-cycle sample reach the highest photocatalytic activity. This optimum photocatalytic performance shows around 3200-fold improvement in degradation rate compared with bare TiO_2_ in the 60 min degradation process under visible-light irradiation. Photocatalytic activities of samples with various cycles increase before reaching the optimum amount then decrease to a lower level. This result is also in agreement with the conclusion of photoelectrochemical properties, which confirms the previous interpretation for the relationship between the amount of Ag_2_S and photoelectrochemical performance. Electrochemical activity reaches optimum value at the 35-cycle sample, while photocatalytic activity reached the maximum value at the 25-cycle sample, indicating the performance of the photocatalyst in a real pollutant degradation reaction depends on multiple factors. Besides, the generation rate and lifetime of photogenerated charge carriers may be related to other factors such as surface area of the sample, particle size of the sensitizer, phase composition and reaction mechanism.

## Conclusions

In summary, TiO_2_ NRAs were prepared by the electron beam deposition with GLAD technique and successive annealing Ti NRAs. Using the SILAR method, we obtained Ag_2_S-coupled TiO_2_ NRAs, with various amounts of Ag_2_S readily controlled by reaction cycle numbers.

By depositing Ag_2_S on TiO_2_ NRAs, the visible light response is notably enhanced in UV-visible absorption spectra. Photoelectrochemical and photocatalytic activity has also been remarkably improved under visible light irradiation and showed a significant variation with different reaction cycles. The 35-cycle Ag_2_S-coupled TiO_2_ NRAs reaches the maximum photocurrent density. The optimum photocatalytic rate appears on 25-cycle sample, which exhibits a notable visible-light-induced photocatalytic around 3200-fold improvement compared with bare TiO_2_ NRAs in the 60 min MO degradation process. This method which can utilize visible light resources more efficiently provides a promising candidate for photocatalysis in the application of wastewater treatment and organic pollutant degradation.

To improve the photocatalytic performance of this binary system, prospective researches could be considered including modifying treatment such as ion irradiation^32^, (rapid) thermal annealing^26^,^33^, strong magnetic fields^34^ and their synergy effect^35^. Moreover, promising techniques such as ion implantation^36^ and light etching^37^ induced patterning could be used to control the substrate for synthesizing NRAs. Also, apart from the SILAR method in this study, other surface deposition techniques (such as magnetron sputtering^38^ and chemical vapor deposition^39^) could be applied to introduce Ag_2_S NPs on TiO_2_ NRAs.

## Methods

### Synthesis of TiO_2_ NRAs

The TiO_2_ NRAs were prepared by oxidation of Ti NRAs via annealing. The Ti NRAs were deposited by electron beam deposition using GLAD technique^40^, on quartz, silicon wafer and F-doped SnO_2_ (FTO) substrates, respectively. Quartz substrates were used for UV-vis transmittance measurement, silicon wafer for SEM observation and FTO for photo electrochemical measurement. The quartz, silicon wafer and FTO substrates were successively ultrasonically cleaned in acetone, alcohol and deionized water for 5 min each. With a base vacuum level of 2 × 10^−8 ^Torr, Ti NRAs were deposited at a rate of 7.5 Å/s, where the thickness was monitored by a quartz crystal microbalance. To prepare vertically aligned Ti NRAs, the substrates rotated at a speed of 10 rpm while the incident beam of Ti flux was set at *ca.* 85° from the normal surface of the substrate. Then the Ti NRAs were annealed in a tube furnace under ambient atmosphere, from room temperature (~20 °C) to 450 °C in 90 minutes, maintained at 450 °C for 120 minutes, followed by furnace cooling to room temperature. Thus, the TiO_2_ NRAs on these three different substrates were obtained.

### Deposition of Ag_2_S nanoparticles on TiO_2_ NRAs

Ag_2_S NPs were deposited on TiO_2_ NRAs via SILAR method at room temperature. Briefly, the TiO_2_ NRAs substrate was first immersed into a 0.05 M AgNO_3_ aqueous solution for 30 s, next rinsed with deionized water, then immersed into a 0.05 M Na_2_S aqueous solution for 30 s, and finally rinsed with deionized water again. This four-step procedure was considered as one SILAR cycle. The amount of Ag_2_S NPs deposited could be accumulated by repeating the SILAR cycle. To obtain a series of samples with different amounts of Ag_2_S NPs deposited on TiO_2_ NRAs, this immersion cycle (*n*) was repeated different times in our work, specifically 5, 10, 15, 20, 25, 30, 35, 40 cycles.

### Characterizations

The morphology of all the Ag_2_S-coupled TiO_2_ NRAs samples was characterized by a field emission scanning electron microscope (SEM JEOL-7001 F). The microstructures of the samples were also characterized with a transmission electron microscope (TEM JEOL-JEM2011). The UV-visible diffuse reflectance spectra (DRS) for the as-prepared samples were investigated using a UV-vis spectrophotometer (PerkinElmer Lambda 35).

### Photoelectrochemical properties

Photoelectrochemical properties were investigated by measuring the photocurrent intensity versus potential (*I-V* curve) using an electrochemistry workstation (CHI 660d, Chenhua Instrument). These measurements were carried out in a 250 mL quartz cell using a standard three-electrode configuration, composed of the samples on FTO substrates as a working electrode, a Pt foil as a counter electrode, a saturated Ag/AgCl as a reference electrode, and 1 M Na_2_S aqueous solution as the electrolyte. The working electrode was illuminated by a 300 W Xe lamp with power of ~130 mW/cm^2^. An ultraviolet cutoff filter was inserted between the light source and the quartz cell to exclude UV light with a wavelength below 420 nm.

### Photocatalytic properties

To evaluate the photocatalytic performance of samples, the photodegradation reactions of MO were performed. The decomposition of MO with the as-prepared Ag_2_S-coupled TiO_2_ NRAs was examined by its optical absorption spectroscopy. In typical photodegradation reactions, samples with uniformly-sized quartz substrates (15 mm × 15 mm) were added into 5.0 mL MO aqueous solution (1 mM) in a 10 mL beaker one at a time. Then, the system was placed in a petri dish filled with cooling water, and illuminated by the 300 W Xe lamp for 20 and 60 min, respectively, with a filter cutting off light with the wavelength below 420 nm. Before, during and after the reactions, a solution of 3.0 ml MO was drawn out to measure the concentration of MO using a UV-vis spectrophotometer (PerkinElmer Lambda 35). To differentiate the evaporation effect on the concentration of MO, a blank control system of 5.0 mL MO without photocatalytic samples was introduced during the degradation process.

## Additional Information

**How to cite this article**: Li, Z. *et al*. Role of Ag_2_S coupling on enhancing the visible-light-induced catalytic property of TiO_2_ nanorod arrays. *Sci. Rep.* **6**, 19754; doi: 10.1038/srep19754 (2016).

## Figures and Tables

**Table 1:** Parameters obtained from the photocurrent density-voltage curves of TiO_2_ NRAs and Ag_2_S-coupled TiO_2_ for various cycles.

Cycles	*J*_sc_(mA/cm^2^)	*V*_oc_(V)	*η* (%)
0	0.062	0.420	0.002
5	1.227	0.568	0.006
10	1.343	0.623	0.017
15	1.112	0.620	0.015
20	1.547	0.645	0.017
25	2.844	0.687	0.034
30	4.243	0.699	0.046
35	6.256	0.707	0.054
40	0.302	0.692	0.019
